# Effect of heat treatment and nitrogen atmosphere during post-curing on mechanical properties of 3D-printed orthodontic aligners

**DOI:** 10.1093/ejo/cjad074

**Published:** 2023-12-11

**Authors:** Mélanie Mattle, Spiros Zinelis, Georgios Polychronis, Olga Makou, Nearchos Panayi, Spyridon N Papageorgiou, Theodore Eliades

**Affiliations:** Clinic of Orthodontics and Pediatric Dentistry, Center of Dental Medicine, University of Zurich, Zurich, Switzerland; Department of Biomaterials, School of Dentistry, National and Kapodistrian University of Athens, Athens, Greece; Department of Biomaterials, School of Dentistry, National and Kapodistrian University of Athens, Athens, Greece; Department of Biomaterials, School of Dentistry, National and Kapodistrian University of Athens, Athens, Greece; Clinic of Orthodontics and Pediatric Dentistry, Center of Dental Medicine, University of Zurich, Zurich, Switzerland; Department of Dentistry, European University Cyprus, Nicosia, Cyprus; Clinic of Orthodontics and Pediatric Dentistry, Center of Dental Medicine, University of Zurich, Zurich, Switzerland; Clinic of Orthodontics and Pediatric Dentistry, Center of Dental Medicine, University of Zurich, Zurich, Switzerland

**Keywords:** orthodontic, aligners, 3D printing, FTIR, mechanical properties, instrumented indentation testing

## Abstract

**Objectives:**

Three-dimensional (3D)-printed aligners present a promising orthodontic treatment modality, whose clinical success largely depends on the material’s mechanical properties. The aim of this study was to evaluate the mechanical properties of resin-made 3D-printed aligners and assess the effect of two different post-curing conditions.

**Materials and methods:**

Forty dumbbell-shaped specimens and 40 resin aligners were 3D-printed and divided into four equal groups according to post-curing conditions: presence or absence of oxygen during post-curing and water heat treatment at 85°C for 15 s or none. Samples from the central incisor of the aligner (*n* = 5/group) were studied by Attenuated Total Reflection Fourier-transform infrared spectroscopy (ATR-FTIR). The dumbbell-shaped specimens were loaded up to fracture under tensile mode and yield strength, ultimate tensile strength, elastic and plastic strain were calculated. The first mandibular molar area from 3D-printed aligners (*n* = 10/group) was cut and embedded in acrylic resin and then underwent metallographic grinding and polishing followed by instrumented indentation testing to determine the following mechanical properties: Martens hardness, indentation modulus, elastic index, and indentation relaxation. After descriptive statistics, differences according to each post-curing protocol, as well as their combination, were analyzed with linear regression modeling at a 5% significance level.

**Results:**

All groups showed identical ATR-FTIR spectra, while no statistically significant effects were seen for either post-curing protocol (N_2_ presence and heat treatment) or their combination (*P* > .05 in all instances).

**Conclusions:**

The mechanical properties of 3D-printed resin aligners were not considerably affected either by post-curing in N_2_ atmosphere or heat treatment.

## Introduction

Since their introduction in the early 90s, aligners have gathered considerable interest from patients and are today an attractive treatment option for adults with high aesthetic demands [1, 2]. The recent introduction of 3D printing technology in dentistry has enabled clinicians to produce appliances in the office, thereby reducing costs and delivery time, while at the same time being able to satisfy the increasing demand and having a greater say in the design of the provided appliances. This computer-aided additive appliance manufacturing technique utilizes digitized three-dimensional (3D) models and fabricates the orthodontic aligners in subsequent printed layers [3]. Various printing methods exist (like selective laser melting, inkjet printing, and extrusion printing), and stereolithography, along with liquid crystal display and digital light processing, is extensively used for orthodontic appliances [4–9]. All these techniques use a high-intensity light source to partially cure the aligner material in an incremental stratified fashion [10, 11]. In contrast to the polyethylene terephthalate glycol and polyurethane aligners produced by thermoforming on existing models, the majority of 3D-printed aligners are made of a liquid resin being polymerized in a malleable-soft form by the light source [3]. This is followed by a post-curing process in special light polymerization chambers, which further solidifies the aligners and grants them their final mechanical properties. The latter are of paramount importance for the aligner’s clinical efficiency, and therefore, fabrication conditions should be streamlined as much as possible.

In particular, the oxygen inhibition zone is a well-studied resin polymerization hindering phenomenon for curing processes taking place under an open atmosphere [12]. Since 3D-printed aligners are constructed in a layer-upon-layer fashion, exposure to air during material setting may affect the degree of double bond conversion [13] and thus the presence of residual uncured free resin monomer, compromising the mechanical properties of the aligner. Several studies have indicated that nitrogen-containing post-curing chambers free of oxygen have an effect on mechanical properties like modulus of elasticity, hardness, and surface roughness [13–15]. These may have detrimental effects on the magnitude of force applied by the aligner, its relaxation behavior, its aesthetics, and its resistance to wear [16]. Additionally, the intraoral release of the uncured substances from orthodontic materials may have cytotoxic implications and should, therefore, be thoroughly investigated [17, 18].

Furthermore, the additive manufacturing technique, despite being cost-effective, is known to introduce stresses between the printed material layers in an anisotropic manner. This phenomenon is a well-studied side-effect when metallic components are constructed the same way [19] and is partially addressed by post-printing thermal treatment. These residual stresses are not to be taken lightly, since they may have implications on the mechanical properties of the material that might affect the clinical performance of the end-product. In addition, heat applied to the printed aligner might, apart from its described compensatory role, also augment resin monomer conversion rate, polymerization reaction, and degree of cross-linking [20]. Immersion in a hot water bath may also facilitate the release of residual monomers and loosely bound molecules, reducing the risk of any biological consequences by intraoral monomer release of such polymeric devices. However, experimental data supporting these potential advantages are not known to the authors.

The aim of the present study was to investigate the effect of oxygen-free post-curing environment and heat treatment during post-curing on the mechanical properties of 3D-printed resin aligners. The null hypothesis was that there is no statistical difference in the mechanical properties of 3D-printed aligners between a Nitrogen gas atmosphere and an oxygen atmosphere in post-curing or between heat-treated end-product and non-treated aligner during post-curing.

## Materials and methods

### Sample preparation

Forty dumbbell-shaped specimens and forty 3D-printed aligners were fabricated, both from a resin indicated for 3D printing of orthodontic aligners (Tera Harz TC-85DAC, Graphy, Seoul, Korea), using the SprintRay Pro 95 (SprintRay, Los Angeles, CA, USA) via direct light processing technology and equally shared in four groups: (i) standard group, where aligners were treated for 14 min in a post-curing unit (Tera Harz Cure 2, Graphy, Seoul, Korea); (ii) standard treatment plus nitrogen treatment, where the chamber of post-curing unit was purged with Ν_2_ during post-curing polymerization; (iii) standard treatment plus heat treatment, where aligners were additionally immersed in hot water (85°C) for 15 seconds; and (iv) where both nitrogen and heat treatments were added to the standard protocol. All of them were printed in successive layers of 100 μm nominal size employing a 405 nm blue-violet light and digital light processing technology. A centrifugation machine was used to remove excess resin for 4 min. The dumbbell specimens were printed according to the ISO 527-2:2012 standard (1BA specimen type, overall length 75 mm, thickness, and width at narrow portion 2 mm and 5 mm, respectively, and gauge length equal to the length of narrow parallel-sided portion 30 mm). The aligners were printed with a 0.5 mm thickness and 0.05 mm offset from the teeth.

### Attenuated Total Reflection (ATR) - Fourier-Transform InfraRed (FTIR) spectroscopy

Five aligners from each group were sectioned in the middle, and samples from the aligner area corresponding to the central incisor from all groups were cut with a lancet. Each sample was placed against the diamond reflective element of a single-reflection ATR accessory equipped with Zn-Se lenses (Golden Gate, Specac, Kent, UK). The sample was pressed with the sapphire anvil of the accessory to achieve firm contact. ATR-FTIR spectra were acquired from each sample using an FTIR spectrometer (Spectrum GX, Perkin-Elmer Corp, Bacon, UK) operated under the following conditions: 4000–650 cm^−1^ range, 4 cm^−1^ resolution, and 20 scan co-addition. The depth of analysis was estimated as ~2 μm at 1000 cm^−1^. All spectra were subjected to ATR and baseline corrections, while aliquots of unset resin were also analyzed to estimate the degree of conversion of double bonds.

### Tensile testing

After measuring the dimensions of each dumbbell-shaped specimen with a digital micrometer (Mitutoyo, Kanagawa, Japan), all specimens were loaded up to fracture with a universal tensile testing machine at a crosshead speed of 10 mm/min speed (Tensometer 10, Monsanto, Swidon, UK). Specimens were mounted in mechanical wedge action tensile grips which are dedicated to a wide range of materials (metallic materials, composites, etc.) and specimen types. These grips are designed to provide a manual wedge action grip, facilitating positioning and alignment of tested specimens and eliminating specimen slippage during loading. In addition, stress was calculated by dividing the measured force by the nominal cross-section area and strain by dividing the disposition of the cross head from zero position divided by the nominal gauge length and multiplied by 100%. The yield strength (YS) and the ultimate tensile strength (UTS) were calculated by dividing the force by nominal cross-section area of each specimen. YS was defined as the point of the curve where the slope of the tensile curve changes significantly or as the highest point of the first local peak after the elastic part of the tensile curve. The strain at the yield strength was considered the elastic strain, while plastic strain was calculated by subtracting elastic strain from total strain according to the ISO 527-1:2012 standard.

### Instrumented indentation testing

The aligner area pertaining to the mandibular first molars from each group was cut from the aligners, and the specimens were embedded in acrylic resin (Verso Cit-2, Struers, Ballerup, Denmark). Samples were oriented during embedding in resin with their occlusal surfaces parallel to the horizontal plane so that the full-thickness cross-section of the aligner’s cervical wall area faced upwards and was used during instrumented indentation testing (IIT). Then, the samples were ground up to 4000 grit-size Si-C papers under water cooling and polished with a water-based diamond suspension (NapR1 DiaPro, Struers, Ballerup, Denmark) of 1 μm particle size in a grinding/polishing machine (Dap-V, Struers, Ballerup, Denmark). The mechanical properties of the four groups were investigated by means of IIT, including Martens Hardness (HM), indentation modulus (E_IT_), elastic index (η_IT_), and indentation relaxation (R_IT_). Testing was conducted in a universal hardness testing machine (ZHU0.2/Z2.5, Zwick Roell, Ulm, Germany) with a Vickers indenter, applying two different loading regimes: (i) the HM, E_IT_, and η_IT_ were determined automatically from force-indentation depth curves applying a maximum load of 4.9N for a 2-s contact time; (ii) for R_IT_, a rectangular force pulse with a constant indentation depth of 50 μm was applied for 120 s and the R_IT_ value was measured by monitoring the decrease in force between the start and the end of the constant indentation depth period. Relaxation was determined by the following equation:


$$\begin{array}{l} R_{IT}\left(\%\right)=100*\left(F1-F2\right)/F1 \end{array}$$

where *F*1 and *F*2 stand for the force at *t* = 0 and *t* = 120 s of rectangular pulse. All mechanical properties were calculated according to formulas provided by the international standards [21–23] using a Poisson’s ratio of 0.357 [24].

### Statistical analysis

Initially, normality was assessed through visual inspection of distribution plots and formally with the Shapiro-Wilk test. Descriptive statistics, consisting of means and standard deviations (SD) for normally distributed data (and medians with inter-quartile ranges [IQR] for skewed data) were calculated. The effect of heat treatment and the effect of N_2_ presence during post-curing were assessed through linear regression models, after checking appropriate regression diagnostics (residuals’ normality, homoscedasticity, and model specification). Initially, an interaction term of heat by N_2_ was introduced in the model and was ultimately dropped from the model if statistically non-significant. All analyses were done in Stata (version 14.0; Stata Corp, College Station, TX) with α = 5% and an openly provided dataset (doi: 10.5281/zenodo.8346723).

## Results

**Figure 1.**
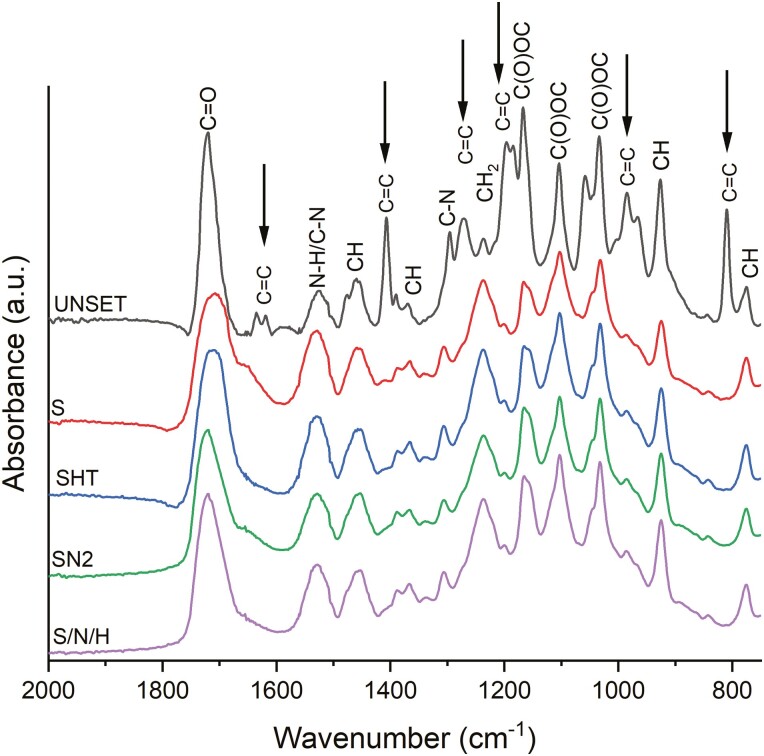
Representative ATR-FTIR spectra from unset material and all groups tested. All groups indicate identical ATR-FTIR spectra. Vertical arrows indicate the C=C in unset material. S, standard group; SHT, standard plus heat treatment group; SN2, standard plus nitrogen treatment group; S/N/H, standard plus nitrogen and heat treatment group.

Figure 1 illustrates representative ATR-FTIR spectra from the four groups tested and samples of unset resin. The spectra from all groups tested are identical and present the characteristic bands of NH, CH, C=O, CN, C(O)OC, and COC groups, which fit with a polyester–urethane polymer structure. All peaks of C=C (indicated by arrows) of unset material vanished after setting, denoting a complete conversation of double bonds.

**Figure 2.**
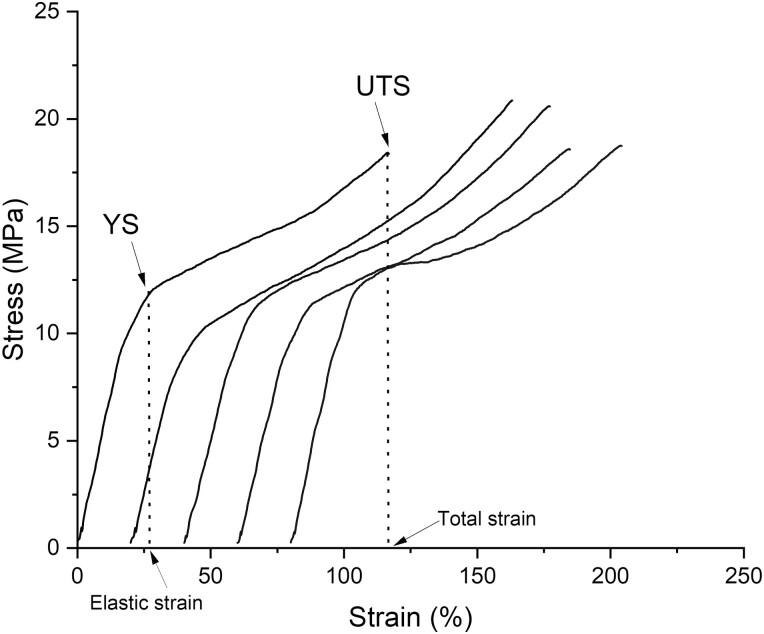
A set of representative stress-strain curves from all groups tested under tensile testing of the dumbbell-shaped specimens. YS and UTS stand for yield strength and ultimate tensile strength, respectively. Elastic and total strain are also indicated by arrows on horizontal axis. For the sake of clarity, the curves are offset by 20%.

Figure 2 indicates representative stress–strain curves from all groups tested. Characteristic points of YS, UTS, and elastic and total strain are indicated by arrows on the first curve. The curves are shifted horizontally for the sake of clarity. The results of mechanical properties along with all statistical comparisons, are given in Table 1.

**Table 1.** Descriptive statistics for all mechanical properties tested along with the effect of heat treatment, N_2_ treatment, or their interaction.

Variable	Group	Metric	no N_2_	N_2_	Interaction	HT	N_2_
Yield strength (YS; MPa)	Νο HT	Mean (SD)	11.7 (1.8)	11.6 (1.2)	0.74	0.44	0.90
	HT	Mean (SD)	12.0 (1.3)	12.2 (2.0)			
Ultimate tensile strength (UTS; MPa)	Νο HT	Mean (SD)	20.7 (1.1)	20.8 (1.2)	0.25	0.95	0.20
	HT	Mean (SD)	20.3 (1.5)	21.2 (0.9)			
Elastic strain (%)	Νο HT	Mean (SD)	28.7 (3.1)	26.9 (1.5)	0.31	0.51	0.26
	HT	Mean (SD)	28.4 (1.4)	28.3 (3.7)			
Plastic strain (%)	Νο HT	Median (IQR)	109.5 (103.0–114.0)	113.5 (94.0–112.0)	0.80	0.06	0.24
	HT	Median (IQR)	104.5 (94.0–112.0)	107.0 (101.0–112.0)			
Martens Hardness HM (N/mm^2^)	Νο HT	Median (IQR)	91.0 (86.0–92.0)	91.0 (89.0–93.0)	0.15	0.08	0.43
	HT	Median (IQR)	91.0 (89.0–93.0)	92.0 (90.0–93.0)			
Indentation Modulus E_IT_ (MPa)	Νο HT	Median (IQR)	2883.0 (2802.9–2978.1)	2795.6 (2765.5–2815.3)	0.68	0.59	0.06
	HT	Median (IQR)	2857.7 (2792.6–2921.7)	2759.4 (2725.5–2809.2)			
Elastic index (η_IT_; %)	Νο HT	Median (IQR)	27.8 (27.6–29.1)	28.3 (27.6–29.1)	0.86	0.46	0.14
	HT	Median (IQR)	27.8 (27.3–28.7)	28.5 (28.3–28.8)			
Relaxation index R_IT_ (%)	Νο HT	Mean (SD)	60.3 (1.7)	57.8 (1.8)	0.22	0.46	0.09
	HT	Mean (SD)	58.6 (4.0)	58.2 (2.8)			

HT, heat treatment; IQR, interquartile range; SD, standard deviation.

**Figure 3.**
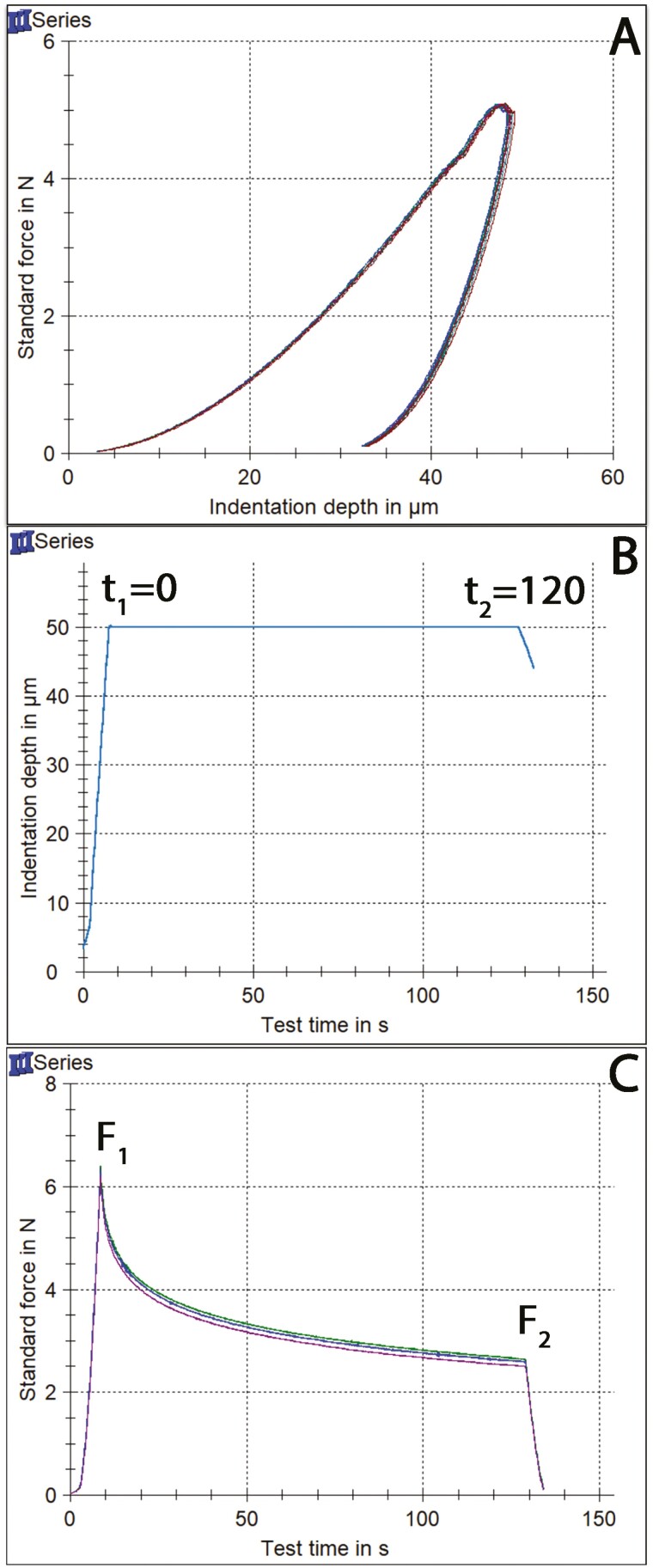
(A) Force indentation depth curves from all groups tested. (B) The rectangular pulse of standard indentation depth of 50 μm for 120 s applied for the measurement of relaxation index. (C) Representative curves of force decay over time of material tested during application of rectangular pulse.

Figure 3A illustrates representative force-indentation depth curves for all groups tested. Figure 3B presents the rectangular pulse applied with a period of standard indentation depth, while Fig. 3C depicts force degradation over recording time. All groups show similar indentation depth, as no differences were identified in HM.

All mechanical properties determined by both tensile and IIT measurements are shown in Table 1 along with statistical comparisons according to the presence of oxygen and the heat treatment during the aligners’ post-curing. No significant differences were found for all mechanical properties tested either between groups post-cured with and without N_2_ atmosphere or heat treatment (*P* > .05 in all instances). The average material properties among all specimens tested (*n* = 40) were as follows: YS mean 11.9 MPa (SD 1.6 MPa); UTS mean 20.7 MPa (SD 1.2 MPa); elastic strain mean 28.1% (SD 2.6%); plastic strain median 110.5% (IQR 101.0–114.0%); HM median 91.0 N/mm^2^ (IQR 89.0–93.0 N/mm^2^); E_IT_ median 2806.8 MPa (IQR 2746.2–2900.5 MPa); ηIT median 28.3% (IQR 27.4–28.8%); R_IT_ mean 58.7% (SD 2.8%). No statistically significant interaction between N_2_ and heat was identified, which means that the effect of each of these two protocols is independent, and their combination has no effect.

## Discussion

Based on the results of this study, the null hypothesis should be accepted, as no considerable differences in the mechanical properties of the four studied groups were identified.

Spectroscopy results detected no vibrations of aromatic groups, indicating that the used polymer is an aliphatic vinyl-functionalized polyester–urethane material, which is in accordance with previous reports [25]. The C=C peaks of the unset material completely vanished on set groups, indicating full conversion of double bonds in all cases. Therefore, it can be stated that none of the discriminating variables had any beneficial effects on the C=C conversion degree. Spectra of the tested groups indicated identical patterns, with the absence of any sign of compositional differences arising from post-curing in different environmental conditions (either a nitrogen atmosphere or thermal treatment).

Given that the used dumbbell-shaped specimens are bulkier (2 × 5 mm cross section) than orthodontic aligners (ranging from 0.5 to 0.65 mm in thickness), IIT was combined with tensile testing to determine the mechanical properties of orthodontic appliances, excluding the limitation that size could play a significant role on mechanical properties tested. IIT has been extensively used as it is capable of automated, subjective, and rebound-free evaluation of the material end-product [16, 26, 27].

No significant interactions were detected between the independent variables of a nitrogen atmosphere or water-based heat treatment (Table 1), indicating that the effect (or lack of it) from each of the post-curing conditions was independent and no added benefit existed by their combination.

All recorded stress–strain curves demonstrated the typical pattern of polymeric materials’ tensile curves [28], where the yield was followed by an increase in stress up to the material’s final fracture. During this stage, following the yield strength, the polymer chains tend to orient parallel to the tensile vector resulting in a strain-hardening mechanism [29]. Although stressing beyond the yield strength is clinically insignificant, the absence of any significant differences among groups for fracture strength and plastic deformation implies that both treatments did not seem to affect the composition or structure of the tested material. This also verifies the abovementioned findings from the FTIR analysis.

The results of IIT are in full accordance with the results of tensile testing, as no significant differences were seen among the groups for all mechanical properties tested. As far as hardness is concerned, the HM values (median of 91.0 N/mm^2^) suggest a material that is in consensus to earlier studies, equally hard to the thermoplastic polyethylene terephthalate glycol, and inferior to the material used by Invisalign (Align Technology, Santa Clara, CA) [16, 25, 30–34]. The harder the aligner is, the more its resistance to intraoral wear, which is beneficial from the standpoint of structural integrity. The average modulus of elasticity was 2806.8 MPa, which is comparable to previous studies assessing resin-made aligners (2491.2–2696.3 MPa) [16, 25] and the polyurethane Invisalign ones (2216.0–2466.0 MPa) [30, 31], while thermoformed aligners are considerably less stiff (1365.2-2374.0 MPa) [32–34]. A higher modulus of elasticity is beneficial, as it provides increased counterforce for equal strain or equal counterforce for smaller thickness, thereby facilitating patient comfort. The elastic indexes of tested material were, on average, 28.3%, denoting a less brittle behavior compared to Invisalign (40.0–40.8%) and thermoplastic aligners (34.0–35.9%) [30, 31, 33]. A material’s elastic index might influence the risk of fracture due to deformation by the repeated insertion/removal of the aligner many times every day [16, 25, 30–34], although this is of minimal clinical relevance here, as aligners are replaced weekly during orthodontic treatment, and therefore each aligner does not serve for prolonged periods of time. The high relaxation index values measured (58.7% on average) for directly printed aligners indicate an intense relaxation process, which rapidly reaches a plateau phase (Fig. 2C). Certainly, it is not comparable to the extremely low values of Invisalign aligners (~4%) [31].

Thus, both investigated post-curing regimes (or their combination) failed to show any added beneficial effect on the mechanical properties of the material tested. Contrary to what might be expected, post-curing in an N_2_ atmosphere takes place after the initial polymerization of material during 3D printing, and therefore, its effect seems to be minimal. Besides, even in the absence of an N_2_ atmosphere, the material shows a full conversion of double bonds according to the FTIR findings. Heat treatment, also known as annealing, is used primarily to relieve and homogenize the field of residual stresses developed during light curing [35]. Additionally, immersion in hot water may facilitate the release of uncured monomers decreasing the risk of any later monomer release intraorally. Similar to post-curing under an N_2_ atmosphere, the contribution of this procedure is expected to be minimal in a fully polymerized material. However, the results of this study should not be unanimously accepted for all clinical scenarios, as a vast array of different resins is used today for this purpose, and the effect of tested parameters could differ substantially among different materials.

## Conclusions

Under the limitations of this study, the cured material showed a full conversion of double bonds. Neither post-curing in the N_2_ atmosphere nor heat treatment had any added beneficial effect on the mechanical properties of 3D-printed orthodontic aligners.

## Data Availability

The dataset of the present study is openly available (doi:10.5281/zenodo.8346722).
